# A Comparative Study of Stabilities and Coordination Modes of β-Alaninephosphonic Acid in Copper(II) Heteroligand Complexes with Ethylenediamine, Diethylenetriamine or *N*,*N*,*N*′,*N*′,*N*″-Pentamethyldiethylene Triamine in Aqueous Solution

**DOI:** 10.1007/s10953-012-9914-4

**Published:** 2012-11-08

**Authors:** Anna Kamecka

**Affiliations:** Institute of Chemistry, Siedlce University of Natural Sciences and Humanities, 3-Maja 54, 08-110 Siedlce, Poland

**Keywords:** Copper(II) complexes, Heteroligand complexes, Aminophosphonic acids, EPR, Vis spectroscopy, Potentiometry, Equilibria, Stability constants, Polyamines

## Abstract

Solution equilibrium studies on Cu^2+^–L_1_–L_2_ ternary systems have been performed by pH-potentiometry, UV–Vis spectrophotometry and EPR methods {where L_1_ corresponds to a polyamine such as ethylenediamine (en), diethylenetriamine (dien), *N*,*N*,*N*′,*N*′,*N*″-pentamethyldiethylenetriamine (Me_5_dien)} and L_2_ denotes 2-aminoethylphosphonic acid (β-alaninephosphonic acid)}. The results suggest the formation of heteroligand complexes with [Cu(L_1_)(β-Ala(P))] stoichiometry in all of the studied systems. Additionally, in the system with en, [Cu(en)(β-Ala(P))H_−1_]^−^ is formed in basic solutions. Our spectroscopic results indicate tetragonal geometry for the [Cu(en)(β-Ala(P))] species, a geometry slightly deviated from square pyramidal for the [Cu(dien)(β-Ala(P))] complex, and somewhat stronger geometry distortion was present for the [Cu(Me_5_dien)(β-Ala(P))] complex. The coordination modes in these heteroligand complexes are discussed.

## Introduction

Aminoalkylphosphonic acids occupy an important place among the various compounds containing a P–C bond and the amino group because they are analogues of natural amino acids, the ‘building blocks’ of peptides and proteins. Their utilities as enzyme inhibitors, anticancer agents, antibiotics, neuromodulators, plant growth regulators and herbicides, antibacterial compounds, and many other technical and industrial applications such as chelating agents and scale inhibitors have attracted the interests of chemists for a long time [1, 2]. So, it is worthwhile to assemble information on their formation, stability and structure, and the mutual influence of two different ligands bound to the same metal ion. Chemical speciation calculations based on numerical equilibrium data are of extreme importance for potential future developments.

Our research work on heteroligand complexes with some polyamines and aminophosphonic acids such as glycinephosphonic acid (Gly(P)) and α-alaninephosphonic acid (α-Ala(P)), which are analogues of amino acids, have shown that the substitution of the carboxylate group by the phosphonic function distinctly changes the coordination ability of the ligand in ternary systems [3, 4]. The studies have also demonstrated that, in the heteroligand complexes, the geometry around copper(II) is closely related to the type of polyamine, i.e., to the number of nitrogen atoms of the polyamines which are potential coordination sites. As a continuation of the previous studies, the β-derivative of aminophosphonic acid in the ternary systems has been investigated. According to our knowledge, no data exist for the ternary systems with β-aminophosphonic acids. The arrangement of the donor atoms in β-derivatives of aminophosphonic acid allows for the formation of a six-membered chelate ring. Thus, it seems to be interesting to investigate whether such structural arrangement of the ligand affects the coordination geometry of the heteroligand complexes to the same extent as was observed in the ternary systems with an α-derivative of aminophosphonic acid [3, 4], amino acids [5, 6] or aminohydroxamic acids [7–11].

Consequently, the present paper reports studies on the systems Cu^2+^–L_1_L_2_ {when L_1_ = ethylenediamine (en), diethylenetriamine (dien) or *N*,*N*,*N*′,*N*′,*N*″-pentamethyldiethylene triamine (Me_5_dien), and L_2_ = 2-aminoethylphosphonic acid (β-alaninephosphonic acid) (β-Ala(P)} using pH-metric, spectrophotometric, and EPR methods.

Systematic comparisons between different Cu^2+^–L_1_L_2_ ternary system with L_2_ = Gly(P) or α-Ala(P) have been performed. Although these systems exhibit many similarities, some important differences are also found. The differences between the complex-forming properties of aminophosphonates and aminocarboxylates in ternary systems have been explained by differences in basicity, charge and size of the $- {\text{PO}}_{3}^{2 - }$ and –COO^−^ groups.

## Experimental

### Chemicals

**Scheme 1:**
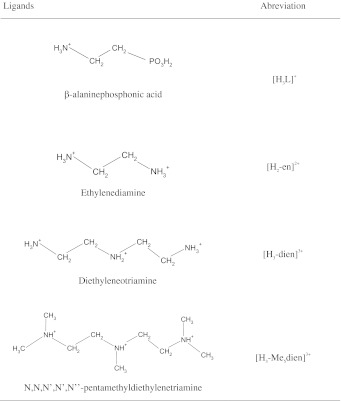
Formulae of the fully protonated forms of the ligands

Ethylenediamine dihydrochloride (en·2HCl), diethylenetriamine, *N*,*N*,*N*′,*N*′,*N*″-pentamethyldiethylene triamine, and 2-aminoethylphosphonic acid were purchased from Sigma-Aldrich Chemical Co. All chemicals were of purity greater than 99 % and they were used without further purification. The purity of the ligands and the concentrations of the ligand stock solutions used for potentiometric measurements were determined by Gran’s method [12]. The CuCl_2_ and HCl standard stock solutions were prepared from Titrisol concentrates (Merck). The exact concentration of copper ions in the stock solution was determined via complexometric ethylenediaminetetraacetate titration. The HCl concentration was determined by pH-potentiometric titrations using Gran’s method [12]. Carbonate-free potassium hydroxide solution (the titrant) was prepared from cc. KOH and standardized against a standard potassium hydrogen phthalate solution. All solutions were prepared with bi-distilled water. The formulae of the fully protonated form of the ligands used in this study are shown in Scheme 1.

### Potentiometric and Spectroscopic Studies

The pH-potentiometric measurements were carried out at an ionic strength of 0.2 mol·dm^−3^ (KCl) at the temperature 25.00 ± 0.1 °C. Carbonate-free KOH solution of known concentration (ca., 0.15 or 0.3 mol·dm^−3^) was used as titrant. A MOLSPIN automatic titrator system equipped with a combination pH electrode (Single Pore Plast or Single Pore Glass, Hamilton) was used for pH-potentiometric measurements. The electrode system was calibrated by periodic titrations of an HCl solution (0.008 mol·dm^−3^ in KCl) against standard KOH solution. The resulting titration data were used to calculate the standard electrode potentials, *E*°, and the dissociation constant for water (p*K*_w_ = 13.74 ± 0.02). These values were then used to calculate the hydrogen ion concentration [H^+^] from the emf readings [13]. All of the pH-potentiometric titrations were performed under an argon atmosphere over the pH range of 2–11 or until precipitation occurred. The initial volume of the samples was 5.00–6.00 cm^3^. For the binary systems the ligand concentration was (2.5–3) × 10^−3 ^mol·dm^−3^ and the copper(II) ion concentrations varied over the range of (0.6–3) × 10^−3 ^mol·dm^−3^ according to the metal ion-to-ligand ratios: 1:1, 1:2 and 1:4. For the ternary systems the ratios of copper(II):en/dien/Me_5_dien:β-Ala(P) were 1:1:1, 1:1:2, 1:2:1, 1:2:2 and 0.5:1:1. Titrations were usually made on samples containing copper(II) ion concentrations of (2.5–3) × 10^−3 ^mol·dm^−3^ or 1.25 × 10^−3 ^mol·dm^−3^ (in the case of the 0.5:1:1 molar ratio). The pH-metric results were utilized in order to find the stoichiometry of the species and to calculate the stability constants of the species formed in the ternary systems. The calculations were made with the aid of the computer program HYPERQUAD 2006 [8]. The equilibrium models for the ternary systems and the corresponding stability constants giving the best fits of the pH-metric titration curves are presented in Table 1.

**Table 1:** Cumulative formation constants (log_10_ *β*), derived equilibrium constants (log_10_ *K*), and characteristic parameters for the stability of complexes formed in the ternary systems Cu^2+^–amine (en, dien or Me_5_dien)–β-alaninephosphonic acid (β-Ala(P)) at 25.00 °C and *I* = 0.2 mol·dm^−3^ (KCl)

Nr	Assignments	System Cu^2+^–L_1_–β-Ala(P); (L_1_ = en, dien or Me_5_dien)		
		Cu^2+^–en–β-Ala(P)	Cu^2+^–dien–β-Ala(P)	Cu^2+^–Me_5_dien–β-Ala(P)
1.	log_10_ *β*[Cu(L_1_)(β-Ala(P))]	17.37 (1)	19.85 (3)	16.59 (1)
2.	log_10_ *β*[Cu(L_1_)(β-Ala(P))H_−1_]^−^	6.17 (2)	–	–
3.	log_10_ $K_{{[{\text{Cu}}\left( {{\text{L}}_{1} } \right)(\beta - {\text{Ala}}\left( {\text{P}} \right))]}}$ ^a^	6.79	3.84	4.12
4.	log_10_ $K_{{\left[ {{\text{Cu}}\left( {{\text{L}}_{1} } \right)\left( {{\text{L}}_{2} } \right)} \right]}}$–${\text{p}}K_{{{\text{NH}}_{3}^{ + } }}$–${\text{p}}K_{{{\text{PO}}_{3} {\text{H}}^{ - } }}$	−10.29	−13.24	−12.96
5.	χ^2^	9.60	11.83	10.88
6.	σ	5.91	6.98	9.98
7.	Δlog_10_ *K* ^b^	−1.47	−4.42	−4.14
8.	log_10_ *X* ^c^	0.47	5.43	–

^a^log_10_ $K_{{[{\text{Cu}}\left( {{\text{L}}_{1} } \right)(\beta - {\text{Ala}}\left( {\text{P}} \right))]}}$ = log_10_ $\beta_{{[{\text{Cu}}\left( {{\text{L}}_{1} } \right)(\beta - {\text{Ala}}\left( {\text{P}} \right))]}}$_ _− log_10_ $\beta_{{\left[ {{\text{Cu}}\left( {{\text{L}}_{1} } \right)} \right]}}$; log_10_ *β*_[Cu(en)]_ = 10.58, log_10_ $\beta_{{\left[ {{\text{Cu}}\left( {\text{en}} \right)_{2} } \right]}}$ = 19.73 [19], log_10_ *β*_[Cu(dien)]_ = 16.01, log_10_ $\beta_{{[{\text{Cu}}\left( {\text{dien}} \right)_{2} ]}}$ = 20.76 [8], log_10_ $\beta_{{\left[ {{\text{Cu}}\left( {{\text{Me}}_{5} {\text{dien}}} \right)} \right]}}$ = 12.47 [9]; log_10_ *β*_[Cu(β-Ala(P))]_ = 8.26, log_10_ $\beta_{{[{\text{Cu}}(\beta - {\text{Ala}}\left( {\text{P}} \right))_{2} ]}}$ = 14.54, ${\text{p}}K_{{{\text{PO}}_{3} {\text{H}}^{ - } }}$ = 6.20 and ${\text{p}}K_{{{\text{NH}}_{3}^{ + } }}$ = 10.88 of β-Ala(P) in this work

^b^ $\Updelta { \log }_{10} K = { \log }_{10} \beta_{{[{\text{Cu(L}}_{ 1} ) (\beta {\text{ - Ala(P}})]}} - ({ \log }_{10} \beta_{{[{\text{Cu(L}}_{ 1} ) ]}} + { \log }_{10} \beta_{{[{\text{Cu(}}\beta {\text{ - Ala(P}})]}} )$, the constant due to the equilibrium: [Cu(L_1_)] + [Cu(β-Ala(P))] ⇆ [Cu(L_1_)(β-Ala(P))] + Cu

^c^ ${ \log }_{10} X = 2{ \log }_{10} \beta_{{[{\text{Cu(L}}_{ 1} ) (\beta {\text{ - Ala(P}})]}} - ({ \log }_{10} \beta_{{[{\text{Cu(L}}_{ 1} )_{2} ]}} + { \log }_{10} \beta_{{[{\text{Cu(}}\beta {\text{ - Ala(P}})_{2} ]}} )$, the constant due to the equilibrium: [Cu(L_1_)_2_] + [Cu(β-Ala(P))_2_] ⇆ 2[Cu(L_1_)(β-Ala(P))]; for the [Cu(Me_5_dien)(β-Ala(P)] heteroligand species the value of this parameter cannot be calculated, since the complex with a 1:2 metal-to-ligand ratio is not formed in the Cu^2+^–Me_5_dien binary system [9]

UV–Vis measurements were performed on the binary and ternary systems. A Beckman DU68 spectrophotometer was used to record the electronic absorption spectra in the range of 300–900 nm at room temperature. The path length was either 1 or 2 cm. The measurements were carried out in aqueous solutions, at different pH values (0.3 pH unit step) between 2.5 and 11, and at all metal-to-ligand ratios studied, with 4 × 10^−3 ^mol·dm^−3^ copper(II) ion concentration. All of the solutions were freshly prepared using bi-distilled water. The metal ion-to-ligand ratios for the copper(II):β-Ala(P) binary system are 1:1 and 1:2. For the ternary systems copper(II):en:β-Ala(P), copper(II):dien:β-Ala(P) and copper(II):Me_5_dien:β-Ala(P), the concentration ratios are 1:1:1 and 1:1:2. These concentration ratios of the reagents were chosen in order to achieve as high as possible concentration of the heteroligand species and to get the lowest concentrations of co-existing species in the solutions. The net ligand-field absorption band of each heteroligand species was extracted from the absorption spectra by applying the procedure described in Ref. [7].

EPR spectra were performed on a Bruker 300E X-band spectrometer equipped with a Bruker NMR gauss meter ER 035 M and a Hewlett-Packard frequency counter HP 5350B at −196 °C and at room temperature. Due to the strong absorption of microwave energy by water, very narrow sample tubes were used for liquid solution measurements. Samples for EPR studies were prepared in water/ethylene glycol (4:1 v/v) solutions to ensure good glass formation with a copper(II) concentration of 4 × 10^−3 ^mol·dm^−3^ in all samples. The copper ion-to-ligands molar ratios are the same as those used in the electronic absorption spectra measurements. The binary and ternary systems solutions were usually studied over the same pH range as in the potentiometric studies. The pH of solutions was measured using a Mettler-Toledo, MP 2300 pH-meter with a combined pH electrode (SINGLE PORE GLASS, Hamilton). The EPR parameters were calculated by computer simulation of the experimental spectra using Bruker’s WIN-EPR SimFonia Software Version 1.25.

## Results and Discussion

The dissociation constants for the ligands and the stability constants of the complexes formed in the binary systems were redetermined under the same experimental conditions as those applied for the ternary systems (ionic strength 0.2 mol·dm^−3^, set with KCl solution). The results are in good agreement with previously published data [8–11, 15–23].

**Fig. 1:**
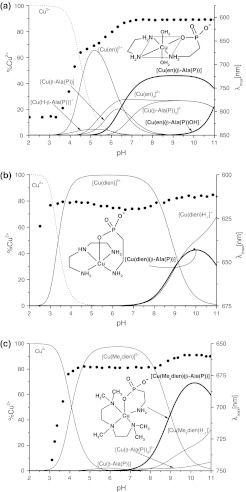
Species distribution curves as a function of pH and variations of the visible absorption maximum wavelength (*circle*) for the Cu^2+^–en–β-Ala(P) system (**a**), Cu^2+^–dien–β-Ala(P) system (**b**), and the Cu^2+^–Me_5_dien–β-Ala(P) system (**c**) at 1:1:2 molar ratio where $c_{{{\text{Cu}}^{2 + } }}$ = 4 × 10^−3^ mol·dm^−3^

The stoichiometries of the complexes and the corresponding stability constants yielding the best fit of the pH-metric titration curves are shown in Table 1. According to these data, heteroligand complexes with [Cu(L_1_)(β-Ala(P))] stoichiometry are formed in all of the studied ternary systems. However, the concentrations of these species in the present systems are distinctly lower than the concentrations of the heteroligand complexes formed in the ternary systems studied by us earlier with the same polyamines and glycinephosphonic acid [3] or with α-alaninephosphonic acid [4]. In the Cu^2+^–en–β-Ala(P) ternary system the hydroxo heteroligand [Cu(en)(β-Ala(P))H_−1_]^−^ species also occur in basic solution. From these results, representative concentration distribution curves calculated at 1:1:2 molar ratios, together with the *λ*_max_ values of the visible spectra as a function of pH for Cu^2+^–en–β-Ala(P), Cu^2+^–dien–β-Ala(P) and Cu^2+^–Me_5_dien–β-Ala(P) ternary systems, are presented in Fig. 1.

The equilibrium constant, $K_{{[{\text{Cu}}\left( {{\text{L}}_{1} } \right)(\beta - {\text{Ala}}\left( {\text{P}} \right))]}}$ (Table 1, row 3), is much lower for the heteroligand species involving the tridentate ligands dien and Me_5_dien than for the species containing the bidentate ligand en. Analogous results were obtained for ternary systems of the same polyamines and phosphonic acid [3, 4] and for different amino acids in copper(II) solutions [5, 6].

Similarly, the obtained value of the Δlog_10_ *K* parameter for [Cu(en)(β-Ala(P))] (Table 1, row 7), which characterizes the tendency towards formation of heteroligand complexes [24], is typical for a complex with distorted octahedral geometry and two different bidentate ligands in the coordination sphere of the Cu^2+^ ion [25, 26]. Values of this parameter close to −1 were obtained for the heteroligand species of tetragonal geometry studied by us earlier, formed by ethylenediamine and other phosphonic acids [3, 4], aminoacids [5, 6], and aminohydroxamic acids in copper(II) solutions [11]. However, in the case of the heteroligand complexes [Cu(L_1_)(β-Ala(P))] with dien or Me_5_dien (Table 1, row 7), the values of Δlog_10_ *K* are clearly lower, most probably because of the lower (than six) coordination number of Cu^2+^. Such low values of this parameter were found previously in the case of five-coordinate copper(II) heteroligand complexes formed with diethylenetriamine or *N*,*N*,*N*′,*N′*,*N″*-pentamethyldiethylene triamine with phosphonic acids [3, 4], as well as aminoacids or aminohydroxamic acids [5–8, 10, 23].

In turn, the value of the log_10_ *X* parameter calculated for the studied heteroligand species (Table 1 row 8) is somewhat lower for [Cu(en)(β-Ala(P))] and distinctly higher for [Cu(dien)(β-Ala(P))] than are expected on a statistical basis (0.6) [24]. This indicates that, in the studied ternary systems, the formation of heteroligand complexes is favored and it is especially preferred in the system with dien. This is a reflection of the ratio of the stepwise formation constants for the binary Cu^2+^–amine system (log_10 _(*K*_Cu(dien)_/$K_{{{\text{Cu}}\left( {\text{dien}} \right)_{2} }}$) = 11.26, which is significantly greater than log_10_ $(K_{{{\text{Cu}}({\text{en}})}} /K_{{{\text{Cu}}({\text{en}})_{2} }} )$ = 1.43. The same effect was observed for the ternary systems with other studied phosphonic acids and amino acids [3–6].

### Cu^2+^–en–β-Ala(P) System

**Fig. 2:**
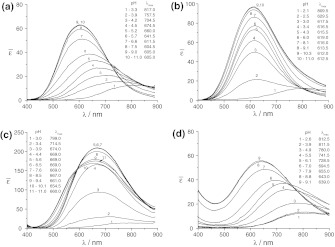
pH-varied absorption spectra of the Cu^2+^–en–β-Ala(P) system (**a**); Cu^2+^–dien–β-Ala(P) system (**b**) at 1:1:2 molar ratio ($c_{{{\text{Cu}}^{2 + } }}$ = 4 × 10^−3 ^mol·dm^−3^, 2 cm cells); Cu^2+^–Me_5_dien–β-Ala(P) system (**c**) at 1:1:2 molar ratio ($c_{{{\text{Cu}}^{2 + } }}$ = 4 × 10^−3 ^mol·dm^−3^, 1 cm cells); and Cu^2+^–β-Ala(P) system at 1:2 molar ratio (**d**) ($c_{{{\text{Cu}}^{2 + } }}$ = 4 × 10^−3 ^mol·dm^−3^, 2 cm cell)

The species distribution curves for the Cu^2+^–en–β-Ala(P) system at 1:1:2 molar ratio as a function of pH, presented in Fig. 1a, indicate that under these experimental conditions [Cu(en)(β-Ala(P))] heteroligand species start to form at higher pH (above pH 5.5) than the analogous heteroligand species formed in the ternary systems with glycinephosphonic (Gly(P)) or α-alaninephosphonic acids (α-Ala(P)) [3, 4]. The EPR parameters of the spectra observed up to this pH in the liquid and frozen solutions (*g*_iso_ = 2.138, *A*_iso_ = 69 × 10^−4^ cm^−1^, *g*_||_ = 2.282, *A*_||_ = 183 × 10^−4 ^cm^−1^) are almost identical to those stated for the Cu^2+^–en binary system in an acid solution [11], which confirms that [Cu(en)]^2+^ predominates. As the pH of the solution increases, a distinct change of the EPR spectral features can be noticed. At pH 6.5 the spectrum typical of the [Cu(en)]^2+^ complex is still observed but a spectrum characteristic of [Cu(en)_2_]^2+^ (*g*_iso_ = 2.101, *A*_iso_ = 84 × 10^−4 ^cm^−1^, *g*_||_ = 2.203, and *A*_||_ = 196 × 10^−4 ^cm^−1^) is also observed. Just above this pH a completely new spectrum is observed for coexisting species in equilibrium. It was obtained by subtraction of the EPR signals from the binary species in equilibrium (with suitable contribution), present also in the Cu–en–β-Ala(P) ternary system over the pH range 8–10. The EPR parameters of this spectrum observed in the liquid and frozen solution at this pH range (*g*_iso_ = 2.117, *A*_iso_ = 77 × 10^−4 ^cm^−1^ and *g*_||_ = 2.237, *A*_||_ = 185 × 10^−4 ^cm^−1^) are completely different from those obtained for the binary systems with β-Ala(P) or en (Tables 2, 3). They should be ascribed to the heteroligand species [Cu(en)(β-Ala(P))] predicted as being dominant in this pH range. The values of the EPR parameters are intermediate between those obtained for the bis(ligand) complexes formed by β-Ala(P) or en (Tables 2, 3) and may be unambiguously assigned to the 3 N donor set, which supports the presence of [Cu(en)(β-Ala(P))] heteroligand species (with NH_2_, NH_2_, NH_2_, ${\text{PO}}_{3}^{2 - }$ donor set). Similarly, the energy of *d*–*d* transition for the [Cu(en)(β-Ala(P))] species, equal to 612 nm, is intermediate between those obtained for the [Cu(β-Ala(P))_2_]^2−^ (642 nm) and [Cu(en)_2_]^2+^ (549 nm) species with 2 N and 4 N coordinations, respectively (Fig. 2a, d; Tables 2, 3). It is easily noticed that the EPR parameters obtained and the absorption spectroscopic data for the studied ternary system are similar to those corresponding to the heteroligand species formed by en and glycinephosphonic or α-alaninephosphonic acid with the same donor atoms in the coordination sphere of copper(II) ions (Table 3) and amino acids [6]. Therefore, they clearly support the formation of (NH_2_, NH_2_) and (NH_2_, ${\text{PO}}_{3}^{2 - }$) chelates in the plane of [Cu(en)(β-Ala(P))]. Above pH 10, a small change in the EPR spectrum may be attributed to a small amount of the next heteroligand species, [Cu(en)(β-Ala(P)H_−1_]^−^.

**Table 2:** Visible and EPR spectral parameters of the complexes formed in the ternary systems Cu^2+^–amine (en, dien or Me_5_dien)–β-alaninephosphonic acid (β-Ala(P))

Species	EPR							Vis
	*g*_iso_	*A*_iso_ (10^−4 ^cm^−1^)	*g*_||_	*A*_||_ (10^−4 ^cm^−1^)	*g*_⊥_	*A*_⊥_ (10^−4 ^cm^−1^)	Donor set	*λ*_max_ (nm) *ε* (dm^3^·mol^−1^·cm^−1^)
[Cu(β-Ala(P))]	2.163	60	2.338	154	2.077	10	{NH_2_, ${\text{PO}}_{3}^{2 - }$}	726.5 (37)
[Cu(β-Ala(P))_2_]^2−^	2.136	64	2.293	165	2.058	14	2{NH_2_, ${\text{PO}}_{3}^{2 - }$}	642 (52)
[Cu(en)(β-Ala(P))]	2.117	77	2.237	185	2.060	17	{NH_2_, NH_2_}; {NH_2_, ${\text{PO}}_{3}^{2 - }$}	612 (85)
	*g*_1_	*g*_2_	*g*_3_	*A*_1_ (10^−4 ^cm^−1^)	*A*_2_ (10^−4 ^cm^−1^)	*A*_3_ (10^−4 ^cm^−1^)		
[Cu(dien)(β-Ala(P))]	2.216	2.057	2.047	198	29	11	{NH_2_, NH, NH_2_}; {NH_2_, ${\text{PO}}_{3}^{2 - }$}	613 (122)
[Cu(Me_5_dien)(β-Ala(P))]	2.225	2.067	2.047	184	31	12	{N(CH_3_)_2_, N(CH_3_), N(CH_3_)_2_}; {NH_2_, ${\text{PO}}_{3}^{2 - }$}	657 (166)

**Table 3:** Comparison of visible and EPR spectral parameters of the copper(II) complexes

Species	EPR							Vis
	*g*_iso_	*A*_iso_ (10^−4 ^cm^−1^)	*g*_||_	*A*_||_ (10^−4 ^cm^−1^)	*g*_⊥_	*A*_⊥_ (10^−4 ^cm^−1^)	Donor set	*λ*_max_ (nm) *ε* (dm^3^·mol^−1^·cm^−1^)
[Cu(Gly(P))] (Ref. [3])	2.160	59	2.330	158	2.071	10	{NH_2_, ${\text{PO}}_{3}^{2 - }$}	755 (35)
[Cu(Gly(P))_2_]^2−^ (Ref. [3])	2.132	66	2.275	164	2.055	14	2{NH_2_, ${\text{PO}}_{3}^{2 - }$}	658 (40)
[Cu(α-Ala(P))] (Ref. [4])	2.158	58	2.328	161	2.070	11	{NH_2_, ${\text{PO}}_{3}^{2 - }$}	723 (39)
[Cu(α-Ala(P))_2_]^2−^ (Ref. [4])	2.130	67	2.271	167	2.055	14	2{NH_2_, ${\text{PO}}_{3}^{2 - }$}	656 (43)
[Cu(en)]^2+^ (Ref. [11])	2.138	68	2.283	183	2.060	14	{NH_2_, NH_2_}	666 (39)
[Cu(en)_2_]^2+^ (Ref. [5, 11])	2.100	86	2.207	198	2.040	20	2 {NH_2_, NH_2_}	549 (71)
[Cu(en)(α-Ala)]^+^ (Ref. [5])			2.230	190	2.051	18	{NH_2_, NH_2_}; {NH_2_, COO^−^}	580 (58)
[Cu(en)(Gly(P))] (Ref. 3])	2.114	77	2.237	191	2.050	19	{NH_2_, NH_2_}; {NH_2_, ${\text{PO}}_{3}^{2 - }$}	597 (54)
[Cu(en)(α-Ala(P))] (Ref [4])	2.114	79	2.235	190	2.050	19	{NH_2_, NH_2_}; {NH_2_, ${\text{PO}}_{3}^{2 - }$}	595 (58)
[Cu(dien)]^2+^ (Ref. [3, 7])	2.114	75	2.234	192	2.055	20	{NH_2_, NH, NH_2_}	614 (78)
	*g*_1_	*g*_2_	*g*_3_	*A*_1_ (10^−4 ^cm^−1^)	*A*_2_ (10^−4 ^cm^−1^)	*A*_3_ (10^−4 ^cm^−1^)		
[Cu(dien)(α-Ala)]^+^ (Ref. [5])	2.217	2.054	2.043	190	30	15	{NH_2_, NH, NH_2_}; {NH_2_, COO^−^}	635 (106)
[Cu(dien)(Gly(P))] (Ref. [3])	2.212	2.054	2.044	199	32	11	{NH_2,_ NH, NH_2_}; {NH_2_, ${\text{PO}}_{3}^{2 - }$}	592 (103)
[Cu(dien)(α-Ala(P))] (Ref [4])	2.211	2.055	2.045	198	31	11	{NH_2_, NH, NH_2_}; {NH_2_, ${\text{PO}}_{3}^{2 - }$}	622 (93)
[Cu(Me_5_dien)]^2+^ (Ref. [23])	2.240	2.065	2.050	183	29	14	{N(CH_3_)_2_, N(CH_3_), N(CH_3_)_2_}	665 (225)
[Cu(Me_5_dien)(α-Ala)]^+^ (Ref. [5])	2.208	2.138	2.007	135	20	62	{N(CH_3_)_2_, N(CH_3_), N(CH_3_)_2_}; {NH_2_, COO^−^}	777 (275)
[Cu(Me_5_dien)(Gly(P))] (Ref. [3])	2.236	2.165	2.01	125	19	66	{N(CH_3_)_2_, N(CH_3_), N(CH_3_)_2_}; {NH_2_, ${\text{PO}}_{3}^{2 - }$}	~827 (231), 750 (sh)
[Cu(Me_5_dien)(α-Ala(P))] (Ref. [4])	2.237	2.160	2.01	128	17	65	{N(CH_3_)_2_, N(CH_3_), N(CH_3_)_2_}; {NH_2_, ${\text{PO}}_{3}^{2 - }$}	~848 (219), 752 (sh)

### Cu^2+^–dien–β-Ala(P) System

**Fig. 3:**
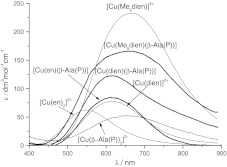
Distribution-corrected absorption spectra for the [Cu(en)(β-Ala(P))], [Cu(dien)(β-Ala(P))], [Cu(Me_5_dien)(β-Ala(P))], [Cu(β-Ala(P))_2_]^2−^, [Cu(en)_2_]^2+^, [Cu(dien)]^2+^, and [Cu(Me_5_dien)]^2+^ species

As is well demonstrated in Fig. 1b, under the applied experimental conditions {Cu^2+^:dien:β-Ala(P) at 1:1:2 molar ratio}, the β-Ala(P) ligand begins to interact with the [Cu(dien)]^2+^ species above pH 7.5. In the pH range 4–7, the EPR spectrum typical for the [Cu(dien)]^2+^ complex (*g*_iso_ = 2.114, *A*_iso_ = 76 × 10^−4 ^cm^−1^ and *g*_||_ = 2.234, *A*_||_ = 194 × 10^−4 ^cm^−1^) is observed and is predominant in the liquid and frozen solutions. Upon increasing the solution pH, a distinct change of the EPR spectral feature can be noticed. The spectrum corresponding to the [Cu(dien)]^2+^ complex is still observed and is dominant between pH = 7.0 and 8.5, but above pH 8.0 a new spectrum is also observed. This new spectrum observed in the frozen solution, with parameters *g*_1_ = 2.216, *g*_2_ = 2.057, *g*_3_ = 2.047 and *A*_1_ = 198, *A*_2_ = 29, *A*_3_ = 11 × 10^−4 ^cm^−1^, that is clearly observed in the pH range 9.0–10.5 in this ternary system, is obviously different from those recorded for the Cu^2+^–dien or Cu^2+^–β-Ala(P) binary systems in this pH range. These results unambiguously show the existence of the heteroligand species [Cu(dien)(β-Ala(P))] in this ternary system. The EPR parameters obtained for this species are consistent with those corresponding to a five-coordinate species formed by dien and other phosphonic acid or amino acids or in the frozen copper(II) solution (Table 3) [3–6]. Therefore, β-Ala(P), similarly to Gly(P) or α-Ala(P), completes the coordination number of five by binding amine nitrogen in the Cu^2+^ plane and phosphonic oxygen in the axial position. This assumption is supported by characteristics of five-coordinate asymmetry in the isolated electronic absorption spectrum obtained for the [Cu(dien)(β-Ala(P))] species over the pH range 9.5–10.5 (Fig. 3) [3–6, 8]. Based on the EPR results (the presence of a weak rhombic distortion, Table 2), it can also be concluded that, similar to other heteroligand species of this type, the geometry of the [Cu(dien)(β-Ala(P))] complex slightly deviates from square pyramidal towards trigonal bipyramidal [27]. Summing up, the structures of this heteroligand species are presented in Fig. 1b and the donor set, {NH_2_, NH, NH_2_}{NH_2_, ${\text{PO}}_{3}^{2 - }$}, is the same as for other heteroligand species formed by the tridentate ligand dien and studied earlier for bidentate phosphonic acids [3, 4].

### Cu^2+^–Me_5_dien–β-Ala(P) System

Representative concentration distribution curves calculated for the Cu^2+^–Me_5_dien–β-Ala(P) system at 1:1:2 molar ratio, together with *λ*_max_ values of the *d*–*d* band, are presented in Fig. 1c. As Fig. 1c shows, up to pH ~ 7.5 the simple species with Me_5_dien is practically the only one present in the solution under these experimental conditions. Simple species with β-Ala(P) are formed in very low concentration (less than 5 %). The EPR parameters of the spectra observed below pH 7.5 in the liquid solution (*g*_iso_ = 2.119, *A*_iso_ = 74 × 10^−4 ^cm^−1^) and frozen solution of this ternary system (*g*_z_ = 2.238, *g*_x_ = 2.067, *g*_y_ = 2.046 and *A*_z_ = 180, *A*_x_ = 24, *A*_y_ = 13 × 10^−4 ^cm^−1^) correspond well to those calculated for [Cu(Me_5_dien)]^2+^ formed in the binary system (Table 3). As the pH of the solution is increased above pH 7.5, heteroligand species start to appear. Both the EPR spectra and absorption spectroscopic data support the existence of the [Cu(Me_5_-dien)(β-Ala(P))] complex (maximum concentration nearly 70 % at pH 9.5–10.5) (Fig. 1c). The EPR spectral characteristics of this species are observed between pH 9.0 and 11.0.

By deconvolution of the EPR spectra recorded in this pH range, the individual spectrum for this complex was calculated. The EPR parameters calculated from the separated EPR spectrum of this heteroligand species in frozen solution, *g*_1_ = 2.225, *g*_2_ = 2.067, *g*_3_ = 2.047 and *A*_1_ = 184, *A*_2_ = 31, and *A*_3_ = 12 × 10^−4 ^cm^−1^, are typical for the “rhombic” spectrum of five-coordinate heteroligand species with a geometry intermediate between trigonal bipyramid and square pyramid [28, 29]. However, the values of *g* parameters and the hyperfine splitting constants *A* of this heteroligand species are different from these obtained for [Cu(Me_5_dien)(Gly(P))], [Cu(Me_5_-dien)(α-Ala(P))] (Table 3), and heteroligand species of amino acids [Cu(Me_5_-dien)(α-Ala)]^+^, [Cu(Me_5_-dien)(Met)]^+^ [6], or [Cu(Me_5_-dien)(l-proline)]^+^ and [Cu(Me_5_-dien)(l-valine)]^+^ [30].

It is very interesting to compare the values of the R {R = (*g*_y _− *g*_z_/(g_x _− g_y_)} parameter calculated for heteroligand complexes formed in the Cu^2+^–L_1_L_2_ ternary systems with L_1_ = dien or Me_5_dien and L_2_ = β-Ala(P) or α-Ala(P). The value of this parameter is a measure of the geometric distortion from square pyramid to trigonal bipyramid and the relative contributions of the $d_{{z^{2} }}$ and $d_{{{\text{x}}^{2} - {\text{y}}^{2} }}$ orbitals in the ground state [29]. For [Cu(-dien)(α-Ala(P))] and [Cu(Me_5_-dien)(α-Ala(P))] the values of R are 0.064 and 1.948 [4], respectively, whereas for [Cu(-dien)(β-Ala(P))] and [Cu(Me_5_-dien)(β-Ala(P))] they are 0.063 and 0.120, respectively. This indicates that the greatest contribution is from the $d_{{{\text{z}}^{2} }}$ orbital in the ground state and a structure closer to a trigonal bipyramid is found when α-Ala(P) and Me_5_dien are involved in the heteroligand complex due to a dominant bulky effect of two methyl substituents at each nitrogen atom. It is worthwhile to mention that a very strong rhombic distortion in the geometry was also observed for phosphonic derivatives of iminodiacetate or nitrilotriacetate complexes, when substitution of the COO^−^ functions by ${\text{PO}}_{3}^{ - 2}$ occurs [31, 32]. It is noteworthy, that for both heteroligand species with dien, the values of this parameter are very small and almost identical and independent of the L_2_ ligand involved. This suggests that the structure of these complexes is much closer to square pyramidal. However in the case of [Cu(Me_5_-dien)(β-Ala(P))] the value of R is significantly lower in comparison with the value of this parameter calculated for [Cu(Me_5_-dien)(α-Ala(P))] and twice as high as the value obtained for heteroligand species with dien. As a conclusion, it can be suggested that the geometric distortion for the [Cu(Me_5_-dien)(β-Ala(P)) species is stronger than for the heteroligand species containing dien (Table 2) but clearly weaker than for [Cu(Me_5_-dien)(α-Ala(P)].

This difference may be assigned to the dissimilar chelate rings formed by both aminophosphonic acids—a five-membered chelate ring in the case of α-Ala(P) and a six-membered chelate ring in the case of β-Ala(P). It should be mentioned here that similar spectroscopic and structural relations between α- and β-derivatives are observed in the case of aminohydroxamic acids [9, 23]. That the [Cu(Me_5_-dien)(β-Ala(P))] complex is dominant between pH 9.5 and 10.5 is also supported by the electronic absorption spectra. The variable pH absorption spectra of the Cu^2+^–Me_5_dien–β-Ala(P) ternary system obtained in basic solutions (Fig. 2c) are different from those for both the Cu^2+^–Me_5_dien [19] and Cu^2+^–β-Ala(P) binary systems (Fig. 2d). The isolated electronic absorption spectrum at pH ~ 10.0 (Fig. 3) exhibits a broad band in the 600–800 nm range with characteristics of five-coordinate complex asymmetry. Moreover, the energy of the *d*–*d* transition for [Cu(Me_5_-dien)(β-Ala(P))] (Fig. 3; Table 2) is consistent with those for five-coordinated heteroligand species with a 4 N donor set [3, 4, 6]. Thus, β-Ala(P) completes the coordination number to five by binding in a bidentate manner (NH_2_, ${\text{PO}}_{3}^{ - 2}$).

#### Stability Comparisons Among Different Cu^2+^–L_1_L_2_ Ternary Systems

It is very interesting to compare the relative stabilities of the complexes formed in the ternary systems with α- and β-derivative of phosphonic acid with those of amino acids (Tables 1, 4). The data clearly show that the relative stabilities of the heteroligand [Cu(L_1_)(L_2_)] species with amino acids [5, 10, 30, 33] are about 2–4 orders of magnitude higher than those for the phosphonic analogues, even though some particular α- and β-derivatives of amino acid and phosphonic acids form chelate rings of the same size and involve a similar mixed-bonding mode (N, O). Accordingly, the relative stability decrease for the heteroligand species of β-Ala(P), which forms six-membered chelate rings, is smaller than those for Gly(P) or α-Ala(P), which form five-membered chelate rings (Tables 1, 4). This can be explained in part by the significantly larger size of the ${\text{PO}}_{3}^{ - 2}$ group. The steric hindrance decreases with increasing size of the chelate ring. The same relative stability decrease effect of phosphonate complexes in comparison to carboxylates was also observed in the case of binary systems with different metal ions [18, 31, 32, 34] and still exists in the ternary system with copper(II) ions. Here is another important point about the relative stability of the heteroligand complexes. The values of the relative stability constants show that the heteroligand complexes formed with Gly(P) or α-Ala(P) are more stable than those formed with β-Ala(P) although the basicity of the coordinating donor groups is higher for the latter ligand. Today we know that the formation of six-membered chelate rings generally results in a decrease of thermodynamic stability and distortion of the coordination geometry of the complexes.

**Table 4:** Comparison of the cumulative formation constants (log_10_ *β*) and derived equilibrium constants (log_10_ *K*) of heteroligand complexes formed in the Cu^2+^–L_1_ (en, dien, Me_5_dien)–L_2_ (L = α-Ala, β-Ala, Gly(P) and α-Ala(P)) systems at 25.00 °C and *I* = 0.2 mol·dm^−3^ (KCl) (charges are omitted for simplicity)

Assignments	α-Ala	β-Ala	Gly(P)	α-Ala(P)
	Ref. [5, 30]	Ref. [10, 33]	Ref. [3]	Ref. [4]
log_10_ *β*[Cu(en)(L_2_)]	17.66	16.58	17.50	17.929
log_10_ *K*[Cu(en)(L_2_)] [Cu(en)] + L_2 _⇌ [Cu(en)(L_2_)]	7.08	6.00	6.92	7.349
log_10_ $K_{{[{\text{Cu}}\left( {\text{en}} \right)({\text{L}}_{2} )]}}$ − ${\text{p}}K_{{{\text{NH}}_{3}^{ + } }}$ − p*K*_COOH_	–4.96	–7.56	–	–
log_10_ $K_{{[{\text{Cu}}\left( {\text{en}} \right)({\text{L}}_{2} )]}}$ − ${\text{p}}K_{{{\text{NH}}_{3}^{ + } }}$ − ${\text{p}}K_{{{\text{PO}}_{3} {\text{H}}^{ - } }}$	–	–	–8.395	–8.2385
log_10_ *β*[Cu(dien)(L_2_)]	20.16	19.41	20.40	20.47
log_10_ *K*[Cu(dien)(L_2_)] [Cu(dien)] + L_2_⇌[Cu(dien)(L_2_)]	4.13	3.40	4.39	4.46
log_10_ $K_{{[{\text{Cu}}\left( {\text{en}} \right)({\text{L}}_{2} )]}}$_ _− ${\text{p}}K_{{{\text{NH}}_{3}^{ + } }}$_ _− p*K*_COOH_	−7.89	−10.23	–	–
log_10_ $K_{{[{\text{Cu}}\left( {\text{en}} \right)({\text{L}}_{2} )]}}$_ _− ${\text{p}}K_{{{\text{NH}}_{3}^{ + } }}$_ _− ${\text{p}}K_{{{\text{PO}}_{3} {\text{H}}^{ - } }}$	–	–	−10.925	−11.1275
log_10_ *β*[Cu(Me_5_dien)(L_2_)]	17.33	15.44	16.286	16.57
log_10_ *K*[Cu(Me_5_dien)(L_2_)] [Cu(Me_5_dien)] + L_2_⇌[Cu(Me_5_dien)(L_2_)]	5.13	3.02	3.816	4.10
log_10_ $K_{{[{\text{Cu}}\left( {{\text{Me}}_{5} {\text{dien}}} \right)({\text{L}}_{2} )]}}$_ _− ${\text{p}}K_{{{\text{NH}}_{3}^{ + } }}$_ _− p*K*_COOH_	−6.89	−10.61	–	–
log_10_ $K_{{[{\text{Cu}}\left( {{\text{Me}}_{5} {\text{dien}}} \right)({\text{L}}_{2} )]}}$_ _− ${\text{p}}K_{{{\text{NH}}_{3}^{ + } }}$_ _− ${\text{p}}K_{{{\text{PO}}_{3} {\text{H}}^{ - } }}$	–	–	−11.499	−11.4875

## Conclusions

In the copper(II)–polyamine-β-Ala(P) ternary systems studied, the potentiometric, VIS and EPR results support the formation of heteroligand complexes with [Cu(L_1_)(β-Ala(P))] stoichiometry. In the case of the [Cu(en)(β-Ala(P))] species, which is present in basic solutions, the formation of (NH_2_, NH_2_) and (NH_2_, ${\text{PO}}_{3}^{ - 2}$) chelates in the equatorial plane of the copper(II) ion is supported. In contrast, complexes of the other heteroligand species with tridentate amines are five-coordinate. For the [Cu(dien)(β-Ala(P))] species, a geometry that deviates slightly from square pyramidal is postulated. In this heteroligand species the binding of Cu^2+^ in the equatorial plane is realized by the three amine nitrogens of dien, and the amine nitrogen of β-Ala(P) and phosphonic oxygen complete the coordination number of five by binding to Cu^2+^ in the axial position. The coordination mode in the heteroligand species containing Me_5_dien is the same as in the case of dien, but the geometric distortion for the [Cu(Me_5_-dien)(β-Ala(P)] species is stronger than for the [Cu(dien)(β-Ala(P))] species. The arrangement of the donor atoms in β-Ala(P) allows the formation of a six-membered (N, O) chelate and results in a decrease of thermodynamic stability and the distortion of the coordination geometry of the complexes, in contrast to the heteroligand species with five-membered chelates such as in the case of the α-derivatives of aminophosphonic acid.

## References

[1] Nowack B (2003). Environmental chemistry of phosphonates. Water Res..

[2] Naydenova ED, Todorov PT, Troev DT (2010). Recent synthesis of aminophosphonic acids as potential biological importance. Amino Acids.

[3] Kamecka A, Kurzak B, Jezierska J, Woźna A, Broda M (2010). Stabilities and coordination modes of glycinephosphonic acid in copper(II) heteroligand complexes with ethylenediamine, diethylenetriamine or *N*,*N*,*N*′,*N*′,*N*″-pentamethyldiethylene triamine in aqueous solution. Struct. Chem..

[4] Kamecka A, Kurzak B, Jezierska J, Woźna A (2011). Stabilities and coordination modes of α-alaninephosphonic acid in copper(II) heteroligand complexes with ethylenediamine, diethylenetriamine or *N*,*N*,*N*′,*N*′,*N*″-pentamethyldiethylene triamine in aqueous solution. J. Solut. Chem..

[5] Kurzak B, Kamecka A, Bogusz K, Jezierska J (2008). Stabilities and coordination modes of histidine in copper(II) mixed-ligand complexes with ethylenediamine, diethylenetriamine or *N*,*N*,*N*′,*N*′,*N*″-pentamethyldiethylenetriamine in aqueous solution. Polyhedron.

[6] Kurzak B, Kamecka A, Bogusz K, Jezierska J, Woźna A (2009). Stabilities and coordination modes of methionine in copper(II) mixed-ligand complexes with ethylenediamine, diethylenetriamine or *N*,*N*,*N*′,*N*′,*N*″-pentamethyldiethylenetriamine in aqueous solution. Polyhedron.

[7] Kurzak B, Kroczewska D, Jezierska J (1998). Ternary copper(II) complexes with diethylenetriamine and α-(or β-) alaninehydroxamic acids in water solution. Polyhedron.

[8] Kurzak B, Bogusz K, Kroczewska D, Jezierska J (2001). Mixed-ligand copper(II) complexes with diethylenetriamine and histidine- or methioninehydroxamic acids in water solution. Polyhedron.

[9] Kroczewska D, Bogusz K, Kurzak B, Jezierska J (2002). Potentiometric and spectroscopic study of mixed-ligand copper(II) complexes with *N*,*N*,*N*′,*N*′,*N*″-pentamethyldiethylenetriamine and α- (or β-) alaninehydroxamic acids in water solution. Polyhedron.

[10] Kurzak B, Kamecka A, Bogusz K, Jezierska J (2007). Unexpected formation of the copper(II) dinuclear mixed-ligand species in the ternary system of *N*,*N*,*N*′,*N*′,*N*″-pentamethyldiethylenetriamine with methionine- or histidinehydroxamic acids in aqueous solution. Polyhedron.

[11] Kurzak B, Kamecka A, Bogusz K, Jezierska J (2007). Coordination modes of histidine- or methioninehydroxamic acids in copper(II) mixed-ligand complexes with ethylenediamine in aqueous solution. Polyhedron.

[12] Gran, G.: Determination of the equivalent point in potentiometric titrations. Acta Chem. Scand. **4**, 559–577 (1950)

[13] Molina M, Melios C, Tognolli JO, Luchiari LC, Jafelicci M (1979). A simple and accurate evaluation of hydrogen-ion concentrations in aqueous solutions of fixed ionic strength. J. Electroanal. Chem..

[14] Gans P, Sabatini A, Vacca A (1996). Investigation of equilibria in solution. Determination of equilibrium constants with the HYPERQUAD suite of programs. Talanta.

[15] Wozniak M, Nowogrocki G (1978). Acidites et complexes des acides (alkyl- et aminoalkyl-) phosphoniques—I: determination potentiometrique des constantes d’acidite par affinement multiparametrique: prise en compte de l’impurete carbonate. Talanta.

[16] Mohan M, Abbott E (1978). Metal complexes of biologically occurring aminophosphonic acids. J. Coord. Chem..

[17] Odani A, Yamauchi O (1984). Preferential formation of ternary copper(II) complexes involving substituted ethylenediamines and amino acids with an aromatic side chain. Inorg. Chim. Acta.

[18] Kiss T, Balla J, Nagy G, Kozłowski H, Kowalik J (1987). Complexes of aminophosphonates. I. Transition metal complexes of aminophosphonic acid analogues of α-alanine, β-alanine, phenylalanine and tyrosine. Inorg. Chim. Acta.

[19] Kurzak B, Kroczewska D (1995). Potentiometric investigation of ternary complexes of nickel, copper, zinc and cadmium with l-α-alaninehydroxamic acid and ethylenediamine. J. Coord. Chem..

[20] Lomozik L, Bolewski L, Dworczak R (1997). Complex formation in copper(II) ternary systems involving polyamines and diaminocarboxylates studiem by potentiometric and spectroscopic techniques. J. Coord. Chem..

[21] Farkas E, Enyedy EA, Micera G, Garribba E (2000). Coordination modes of hydroxamic acids in copper(II), nickel(II) and zinc(II) mixed-ligand complexes in aqueous solution. Polyhedron.

[22] Gąsowska A, Jastrząb R, Bregier-Jarzębowska R, Lomozik L (2001). Intermolecular and coordination reactions in the system of copper(II) with adenosine 5′-monophosphate or cytidine 5′-monophosphate and triamines. Polyhedron.

[23] Kroczewska D, Kurzak B, Jezierska J (2006). The role of the carboxylic group in the copper(II) mixed-ligand complexes of dl-aspartic acid–β-hydroxamic acid and polyamines. Polyhedron.

[24] Sigel H (1973). Metal Ions in Biological Systems.

[25] Sigel H (1975). Stabilität, Struktur und Reaktivität von ternären Cu^2+^-Komplexen. Angew. Chem..

[26] Sigel H (1975). Ternary Cu^2+^ complexes: stability, structure, and reactivity. Angew. Chem. Int. Ed. Engl..

[27] Azuma N, Kohno Y, Izshizu K, Takakuwa T, Tsuboyama S, Tsuboyama K, Kobayashi K, Sakurai T (1994). Spectroscopic studies on copper(II) complexes of chiral cyclens: [CuN_4_Cl] chromophores varying from square pyramidal to trigonal bipyramidal stereochemistry. Inorg. Chim. Acta.

[28] Bencini A, Bertini I, Gatteschi D, Scozzafava A (1978). Single-crystal ESR spectra of copper(II) complexes with geometries intermediate between a square pyramid and a trigonal bipyramid. Inorg. Chem..

[29] Garribba E, Micera G (2006). The determination of the geometry of Cu(II) complexes: an EPR spectroscopy experiment. J. Chem. Educ..

[30] Murakami T, Murata K, Ishikawa Y (1996). Stabilities and spectral properties of five-coordinate mixed-ligand copper (II) complexes containing *N*,*N*,*N*′,*N*′,*N*″-pentamethyldiethylenetriamine and α-amino acids. Inorg. Chim. Acta.

[31] Jeżowska-Bojczuk, M., Kiss, T., Kozłowski, H., Decock, P., Barycki, J.: A simple and accurate evaluation of hydrogen-ion concentrations in aqueous solutions of fixed ionic strength. Complexes of aminophosphonates. Part 8. Copper(II) complexes of N-(phosphonomethyl)amino acids and related compounds. J. Chem. Soc. Dalton Trans. **6**, 811–817 (1994)

[32] Buglyo P, Kiss T, Dyba M, Jeżowska-Bojczuk M, Kozłowski H, Boushin A (1997). Complexes of aminophosphonates—10. Copper(II) complexes of phosphonic derivatives of iminodiacetate and nitrilotriacetate. Polyhedron.

[33] Schischkova L (1974). Study of complex-formation of gallium(IIL) with ammonium-sulfate and determination of stability constant of obtained complex by ion-exchange method. Compt. Rend. Acad. Bulg. Sci..

[34] Kiss T, Farkas E, Kozłowski H, Kowalik J (1989). Complexes of aminophosphonates. II. Transition metal complexes of aminophosphonic acid analogues of aspartic acid and glutamic acid. Inorg. Chim. Acta.

